# Military Surgery among the Apache Indians

**Published:** 1869-10

**Authors:** E. Andrews

**Affiliations:** Prof. of Principles and Practice of Surgery, Chicago Medical College


ARTICLE XXXVIII.

### MILITARY SURGERY AMONG THE APACHE INDIANS.

BY E. ANDREWS, M.D., Prof. of Principles and Practice of Surgery, Chicago Medical College.

The Apaches of New Mexico and Arizona are the most inveterate nomads, perhaps, in the world. They not only have no agriculture, but they despise even the chase. The only occupations they deem worthy of a man are stealing and fighting, the first being the most honorable. Like other Indians, they have their medicine men, and, from the necessities of their position, they have developed a set of ideas and system of practice in military surgery.

Incantations, and other modes of appeasing the unseen powers, constitute a large portion of the resources of the Apache military surgeons, but they are by no means altogether negligent of material appliances. Their prime idea is that the chief danger of a wound is from the loss of blood, a notion which must have been very near the truth in the days when they only received wounds from knives and arrows. They have no idea of a circulation of the blood, but suppose that each part of the body has its own permanent stock of that fluid; but they recognize that hemorrhage from the head, neck, and breast is more dangerous than that from the extremities. From this prime pathological idea, they have naturally made the. deduction that the chief surgical aim should be to plug the wound, and thus put a stop to the hemorrhage. This pathology and treatment they doubtless established centuries ago, from observations upon the incised wounds made by arrows and knives, and being eminently conservative, they naturally apply the same notions and treatment to the gunshot wounds which they receive from the U. S. troops and white settlers.

The first care of the Apache medicine-man, is to have on the field of battle some fresh boughs of the ash, whose leaves are used in the dressings. Quite as important, also, is a quantity of the mescal root. The latter is a nutritious root, which is roasted and carried with them as food. When it is chewed and the nutritive part extracted, there remains in the mouth a wad of woody fibres, like a plug of coarse tow. The doctor first lays on the wound a fresh ash leaf; he then places on it a plug of the chewed mescal fibres, and thrusts the whole into the wound, a plan which in arrow wounds must often be very useful, but rarely of any benefit in gunshot injuries, as the latter seldom bleed. It would seem that they are not content to leave the tampon quietly in its place, but deem it important to change it frequently. After one of the battles in New Mexico, where some twenty or thirty savages were wounded, the U. S. surgeons found the Apache field hospital, and discovered in it about a bushel of the bloody mescal plugs, which had accumulated from their constant change of dressings. After this process has been continued until they deem the danger of hemorrhage entirely over, the next step is to go deeper, for the first plugs were only pushed into the orifices of the wounds. They now fill the entire track of the arrow or bullet to its extremity, with a tampon which, they allow to remain a short time. Finally, they remove it, and complete the cure by the application of herbs externally. All this treatment is accompanied with a suitable amount of superstitious mummery.

During the past year, Mr. S. D. Phelps, a citizen of Chicago, accompanied the U. S. troops to an attack on one of the Apache villages, which was taken by surprise. In the rush, Mr. Phelps came in contact with the medicine-man, and shot him, and as a matter of course, captured his set of surgical instruments. They consisted of five stones and a sea-shell. The stones were carbonate of lime, and were cut out of pieces of beautiful stalagmite, from some cave or spring, the stone being handsomely striped with black and fawn-colored veins. Four of the stones apparently constituted a set of tamponers. The largest was about five inches long, cylindrical and slightly tapering, and was just about the right size to enter a wound made by an army musket-ball. The others were of successive sizes, smaller, and probably used in the same way on the wounds made by the very small stone arrow-heads, of the Apaches and other tribes of that region. The fifth stone is supposed to be a charm only. It represents in form the Texan armadilla, and the stone is ingeniously cut in such a way that the bands of color show stripes across the back like the rows of scales on the armor of the armadilla. The eyes of the animal are represented by pieces of mother of pearl, set into the stone. The sixth object was a sea-shell, perforated and suspended to the neck by a string. It was probably a charm. When the stones were shown to the squaws captured in the assault, the latter exhibited great emotion, and begged that they might be thrown away, otherwise they said not a single soldier would live to return to his camp.

Mr. Phelps has presented these relics to the Chicago Academy of Science, where they may be seen, with other ethnological curiosities, by those desirous to investigate such subjects.

